# Targeting Residual Inflammatory Risk: A Shifting Paradigm for Atherosclerotic Disease

**DOI:** 10.3389/fcvm.2019.00016

**Published:** 2019-02-28

**Authors:** Aaron W. Aday, Paul M. Ridker

**Affiliations:** ^1^Division of Cardiovascular Medicine, Department of Medicine, Vanderbilt Translational and Clinical Cardiovascular Research Center, Vanderbilt University Medical Center, Nashville, TN, United States; ^2^Divisions of Preventive Medicine and Cardiovascular Medicine, Department of Medicine, Center for Cardiovascular Disease Prevention, Brigham and Women's Hospital, Harvard Medical School, Boston, MA, United States

**Keywords:** vascular inflammation, atherosclerosis, residual risk, prevention, randomized trials

## Abstract

As biologic, epidemiologic, and clinical trial data have demonstrated, inflammation is a key driver of atherosclerosis. Circulating biomarkers of inflammation, including high-sensitivity C-reactive protein (hsCRP) and interleukin-6 (IL-6), are associated with increased risk of cardiovascular events independent of cholesterol and other traditional risk factors. Randomized trials have shown that statins reduce hsCRP, and the magnitude of hsCRP reduction is proportional to the reduction in cardiovascular risk. Additionally, these trials have demonstrated that many individuals remain at increased risk due to persistent elevations in hsCRP despite significant reductions in low-density lipoprotein cholesterol (LDL-C) levels. This “residual inflammatory risk” has increasingly become a viable pharmacologic target. In this review, we summarize the data linking inflammation to atherosclerosis with a particular focus on residual inflammatory risk. Additionally, we detail the results of Canakinumab Anti-inflammatory Thrombosis Outcome Study (CANTOS), which showed that directly reducing inflammation with an IL-1β antagonist reduces cardiovascular event rates independent of LDL-C. These positive data are contrasted with neutral evidence from CIRT in which low-dose methotrexate neither reduced the critical IL-1β to IL-6 to CRP pathway of innate immunity, nor reduced cardiovascular event rates.

## Introduction

Numerous lines of investigation have identified robust links between traditional atherosclerotic risk factors and myocardial infarction (MI), ischemic stroke, and peripheral artery disease. Much of ambulatory medical care in the U.S. focuses on the recognition and modification of these risk factors, including hypertension, diabetes, tobacco use, lipid derangements, diet, and physical activity. Despite these efforts, cardiovascular disease has consistently remained the leading cause of death within the U.S. for the past 100 years (1). As a result, researchers have not only focused their attention on ways to more potently ameliorate these traditional risk factors but also to identify and target new pathways of cardiovascular risk. In this review, we discuss the role of inflammation in cardiovascular disease as well as ongoing efforts to test both existing drugs and novel therapeutics targeting inflammation.

## The Biologic Role of Inflammation in Cardiovascular Disease

Vascular biologists have documented the role of inflammation in atherosclerosis for decades. During atherosclerotic plaque initiation, macrophages release proinflammatory cytokines that activate endothelial cells and recruit leukocytes (2). This causes adhesion of leukocytes to endothelial cells, transmigration of leukocytes to the subendothelial space, and plaque progression (2). Activated macrophages and T-cell lymphocytes reside within atherosclerotic plaques, where they multiply and release additional inflammatory cytokines, thus perpetuating a local inflammatory response (3). During plaque maturation, these activated leukocytes are also responsible for smooth muscle cell proliferation and extracellular matrix deposition (4). Beyond atherosclerotic plaque development, the immune system may also contribute to plaque destabilization and rupture, as seen in MI. For instance, evidence suggests that T-cell lymphocytes induce macrophages to release collagenases, which help degrade fibrous caps and reduce collagen synthesis, thus increasing plaque susceptibility to rupture (5).

## Clinical Evidence for Inflammation in Cardiovascular disease

### C-Reactive Protein

Epidemiologic data also support a link between inflammation and cardiovascular disease. As a measure of inflammation, most clinical studies have relied upon C-reactive protein (CRP). CRP is a circulating acute phase reactant synthesized by hepatocytes under the control of numerous inflammatory cytokines, most notably interleukin-6 (IL-6) (6–8). Additionally, CRP is an attractive biomarker of systemic inflammation because of its long half time and the existence of a clinically-available bioassay (8).

The first data in primary prevention showing a connection between plasma CRP levels and incident cardiovascular disease independent of smoking came from the Physicians' Health Study. This was a randomized, double-blind, placebo-controlled trial of aspirin and beta carotene in the primary prevention of cardiovascular disease and cancer among 22,071 apparently healthy middle-aged men free of cardiovascular disease at baseline (9). Following early termination of the trial due to efficacy in the aspirin arm, it was converted to a prospective epidemiologic cohort, and baseline blood samples were collected from 14,916 of the trial participants (10, 11). Using a high-sensitivity CRP assay high-sensitivity C-reactive protein (hsCRP) in 1,086 study participants, researchers found that men in the highest quartile of hsCRP had a 2.9-fold greater risk for MI (*p* < 0.001) and 1.9-fold greater risk for stroke (*p* = 0.02) compared to men in the lowest quartile, and this risk was independent of traditional cardiovascular risk factors (11). Subsequently, the Women's Health Study, which had a similar design but was restricted to apparently healthy middle-aged women, also tested the association between hsCRP and incident cardiovascular disease. In a nested case-control analysis, there was a 1.5-fold increased risk of a composite endpoint including death from coronary heart disease, MI, stroke, or coronary revascularization for each increase in quartile of plasma hsCRP (12).

On the basis of these and other studies, the Emerging Risk Factors Collaboration performed a meta-analysis of hsCRP in 54 prospective cohorts of more than 160,000 individuals free of cardiovascular disease at baseline, which represented 1.31 million person-years of risk (13). In multivariable-adjusted logistic regression analyses, each standard deviation increase in log-transformed hsCRP was associated with a 1.23-fold increased risk for incident coronary heart disease (95% confidence interval [CI], 1.07–1.42). The group found similar outcomes for ischemic stroke (relative risk 1.32; 95% CI, 1.18–1.49) and vascular-associated death (relative risk 1.34; 95% CI, 1.20–1.50). Within this analysis, the risk conferred by increased hsCRP was comparable to that of increased systolic blood pressure, total cholesterol, or non-high-density lipoprotein cholesterol (non-HDL-C) after mutually adjusting for these measures (13).

The JUPITER trial helped further solidify the link between inflammation and atherosclerotic disease. JUPITER was a double-blind, randomized, placebo-controlled trial of 17,802 men and women free of cardiovascular disease with low levels of low-density lipoprotein cholesterol (LDL-C) (<130 mg/dL) and elevated levels of hsCRP (≥2.0 mg/L) (14). Study participants were randomized to either rosuvastatin 20 mg daily or placebo. The trial was terminated early with a median follow-up of 1.9 years. Despite their modest levels of LDL-C at study enrollment and the short duration of treatment, individuals receiving rosuvastatin experienced a 44% reduced risk of a composite endpoint including MI, stroke, arterial revascularization, hospitalization for unstable angina, or death from cardiovascular causes compared to the placebo group. There were similar reductions in MI (54% reduction), stroke (48% reduction), and all-cause mortality (20% reduction).

### Interleukin-6

Despite showing a link between hsCRP and atherosclerotic disease, JUPITER did not demonstrate a causal relationship between inflammation and future cardiovascular events. Other studies also questioned the causal role of CRP. For instance, in a study of 7 healthy adults, direct infusions of CRP did not cause an upregulation of inflammatory cytokines or other acute phase reactants (15). Additionally, Mendelian randomization studies of genetic polymorphisms associated with increased levels of hsCRP found no associated increased risk of atherosclerotic cardiovascular events in these patients (16–18). As a result, investigators have also examined upstream regulators of CRP, including IL-6.

Within the Physicians' Health Study, IL-6 was strongly associated with incident MI; men in the highest quartile of IL-6 had a 2.3-fold greater risk of MI than those in the lowest quartile (*p* = 0.03) (19). Participants in the Women's Health Study had a nearly identical risk association between the highest quartile of IL-6 and cardiovascular events (relative risk 2.2; 95% CI, 1.1–5.3), although this risk association no longer reached statistical significance after adjusting for other traditional risk factors and circulating biomarkers (12). A meta-analysis of 29 prospective studies found that each standard deviation increase in IL-6 was associated with a 25% increase in non-fatal MI or coronary heart disease-related death (20). Additional prospective studies have shown a positive correlation between plasma IL-6 levels and endothelial dysfunction, (21) subclinical carotid atherosclerosis, (22) and type 2 diabetes mellitus (23). In contrast to CRP, Mendelian randomization studies have demonstrated a link between genetic variation associated with plasma IL-6 levels and coronary events, thus suggesting a causal relationship for the IL-6 pathway (24, 25).

### Interleukin-1

Despite clinical data demonstrating an association between IL-6 and atherosclerosis, IL-6 has little role in the clinical setting due to the lack of a readily-available assay and issues related to biomarker stability and half-life (8). Additionally, numerous other inflammatory biomarkers, including IL-18, matrix metalloproteinase-9, soluble CD40 ligand, and tumor necrosis factor (TNF)-α, are also associated with coronary events, suggesting that modulating further upstream inflammatory targets would be necessary to fully attenuate these inflammatory mediators (20). Within this context, the IL-1 signaling pathway emerged as a prime candidate for drug discovery.

The IL-1 family of proteins includes both IL-1α and IL-1β isoforms, both of which bind the IL-1 receptor. IL-1α primarily resides on the cell surface membrane and is mainly involved in paracrine signaling (26). However, numerous triggers involved in atherosclerosis, including cholesterol crystals, turbulent blood flow, and hypoxia, also activate the NLRP3 inflammasome, which integrates these and other signals to activate IL-1β (8, 26). Once activated, IL-1 causes increased leukocyte recruitment and adhesion to endothelial cells as well as increased vascular smooth muscle cell proliferation (27, 28). IL-1 signaling also leads to upregulation of other inflammatory markers, including IL-6, hsCRP, fibrinogen, and plasminogen activator inhibitor (26). In Mendelian randomization analyses, variants regulating the IL-1 receptor antagonist gene are also associated with hsCRP and IL-6 levels (29).

Both clinical and preclinical data support a link between IL-1β, inflammation, and atherosclerosis. Hereditary periodic fever syndromes, such as Muckle-Wells syndrome and familial cold urticaria, are caused by mutations in inflammasome proteins (30). These rare diseases, characterized by severe, recurrent episodes of systemic inflammation and fever, are also associated with increased levels of IL-1β and hsCRP (8, 31). Variants in the IL-1 receptor antagonist gene have also been associated with lower risk of stent restenosis following percutaneous coronary intervention (32). In a porcine model, IL-1β localizes to the sites of iatrogenic coronary injury (33). Additionally, prolonged direct application of IL-1β to porcine coronary arteries triggers intimal thickening and vasospasm, and these effects are attenuated by treatment with an IL-1β neutralizing antibody (34).

## Residual Cardiovascular Risk

Despite this extensive body of research suggesting a link between inflammation and cardiovascular disease, aggressive lowering of LDL-C remains the primary preventive tool for individuals at high risk given the overwhelming body of literature supporting causality (35, 36). Nonetheless, we have clearly not “cured” atherosclerotic disease, and many patients continue to suffer cardiovascular events despite optimal medical therapy. This occurs not only in real-world practice but also within the controlled settings of clinical trials. For instance, PROVE IT-TIMI 22 randomized 4,162 patients with a recent acute coronary syndrome (ACS) to either high dose atorvastatin or pravastatin; the primary endpoint was death, MI, stroke, unstable angina requiring rehospitalization, or revascularization >30 days after the index event (37). Even in the intensive statin arm, there were 464 occurrences of the primary endpoint with an additional 275 recurrent events despite this group achieving a median on-treatment LDL-C of 62 mg/dL (37, 38). Ongoing signaling through lipid, inflammatory, and other biologic pathways confers additional risk on these patients, and this observation has led to the concept of “residual risk.”

**Figure 1:**
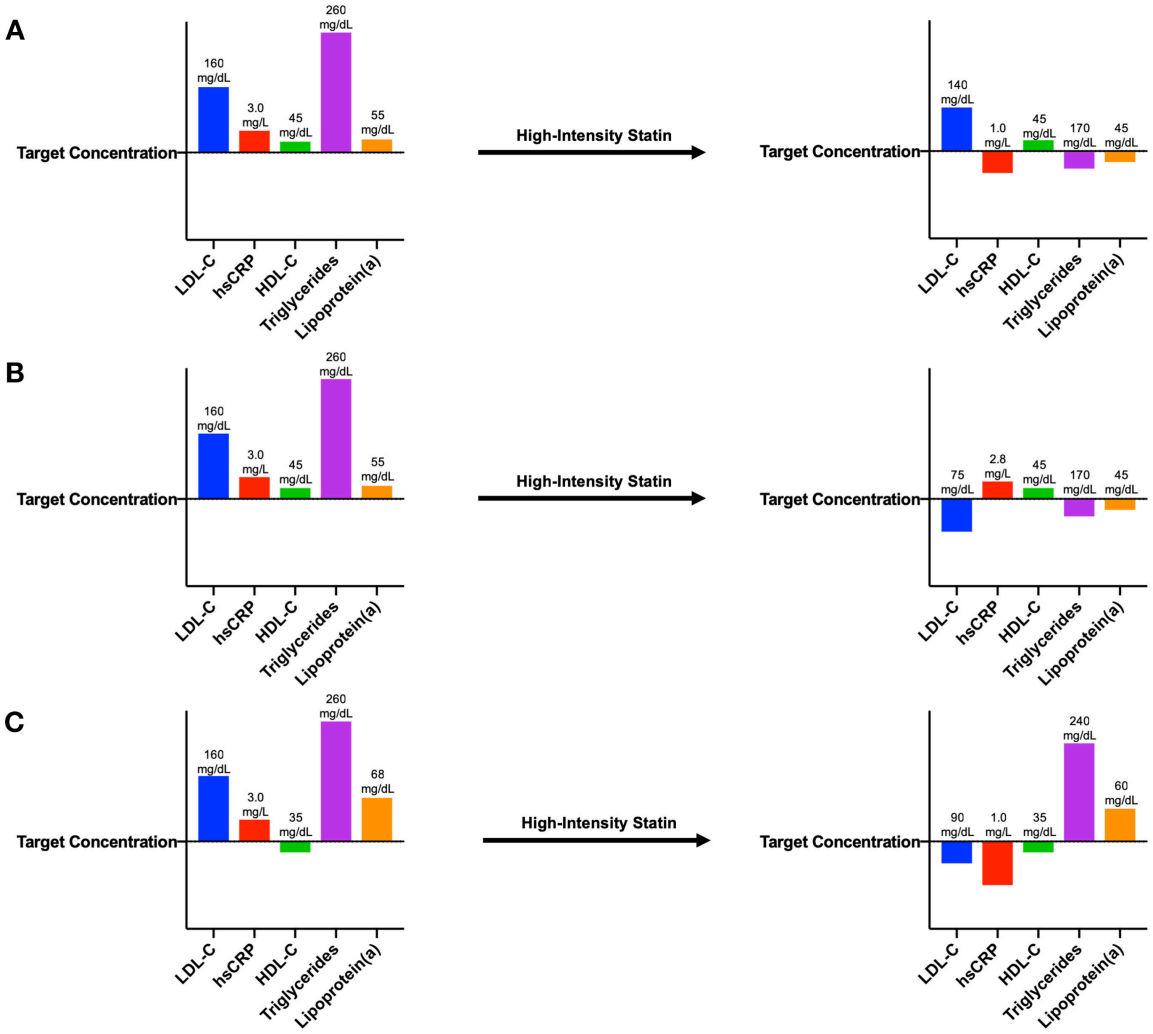
Patient laboratory panels demonstrating various types of residual risk. **(A)** After myocardial infarction, this patient exhibits blood concentrations of LDL-C, hsCRP, triglycerides, and lipoprotein(a) above target concentrations. Following initiation of a high-intensity statin, this individual's LDL-C concentration remains above target, demonstrating residual LDL-C risk. **(B)** After myocardial infarction, this patient also exhibits blood concentrations of LDL-C, hsCRP, triglycerides, and lipoprotein(a) above target concentrations. Following initiation of a high-intensity statin, this individual's hsCRP concentration remains above target, demonstrating residual inflammatory risk. **(C)** After myocardial infarction, this patient also exhibits blood concentrations of LDL-C, hsCRP, triglycerides, and lipoprotein(a) above target concentrations as well as an HDL-C concentration below target. Following initiation of a high-intensity statin, concentrations of HDL-C, triglycerides, and lipoprotein(a) are still not adequately controlled, demonstrating residual risk from these pathways.

Most attempts to address residual risk have focused on further reducing LDL-C using more intensive statins, ezetimibe, or monoclonal antibodies inhibiting proprotein convertase subtilisin-kexin type 9 (PCSK9), and numerous additional drugs in this space are currently under investigation. However, residual risk can be conferred by any molecule or pathway implicated in cardiovascular disease that is not optimally controlled, including HDL-C, serum triglycerides, lipoprotein(a), and inflammation (Figure 1). In some individuals, further lowering of LDL-C may be of limited benefit if inflammation is driving a significant proportion of their residual risk, thus providing the basis for an individualized pharmacologic regimen among individuals at highest risk.

Residual inflammatory risk is not rare, as evidenced by data from several clinical trials. Within PROVE-IT TIMI 22, 29% of trial participants had residual inflammatory risk defined by on-treatment levels of LDL-C <70 mg/dL but hsCRP levels ≥2 mg/L (39, 40). An additional 14% had both residual inflammatory risk and residual cholesterol risk with an LDL-C ≥70 mg/dL. IMPROVE-IT randomized 15,179 patients following ACS to simvastatin 40 mg daily with or without ezetimibe 10 mg daily (41). Similarly, despite achieving average LDL-C levels <70 mg/dL in both groups, 33% had residual inflammatory risk, and 14% had both residual inflammatory and cholesterol risk (40, 42). Finally, in the SPIRE-1/2 trials of the PCSK9 monoclonal antibody bococizumab, which included 9,738 patients with either a history of cardiovascular disease or at high risk for cardiovascular events, 47% of study participants had residual inflammatory risk with or without residual cholesterol risk (43). These observations have led to increasing interest in developing and testing cardiovascular drugs that reduce inflammation (Table 1).

**Table 1:** Anti-Inflammatory drugs targeting residual inflammatory risk in atherosclerotic disease.

**Drug**	**Mechanisms of action**	**Animal data**	**Clinical trial data**
Colchicine	Inhibits microtubule polymerization Prevents activation of NLRP3 inflammasome Reduces release of IL-1β	Reduction in CRP (44)	Reduction in hsCRP, IL-1β, IL-18, IL-6 (45, 46) LoDoCo: reduction in ACS, out-of-hospital arrest, and ischemic stroke LoDoCo2: recruiting COLCOT: recruiting
Allopurinol	Purine analog inhibiting xanthine oxidase	Reduction in aortic atheroma, foam cells, and cytokine release (47)	Reduction in recurrent MI (48) Reduction in cardiovascular events following STEMI (49)
Salsalate	NF-κB inhibitor	NA	TINSAL-T2D: no impact on hsCRP (50) TINSAL-FMD: no impact on flow-mediated dilation (51) TINSAL-CVD: no change in hsCRP or coronary plaque volume (52)
Tocilizumab	Monoclonal antibody against IL-6	NA	Increased clearance of hsCRP following acute NSTEMI (53) No change in coronary flow reserve following NSTEMI (54) ENTRACTE: non-inferior to TNF-α inhibitor in reducing cardiovascular events (55)
Sarilumab	Monoclonal antibody against IL-6	NA	Reduction in hsCRP (56)
Anakinra	Humanized monoclonal antibody against IL-1	NA	Reduction in left ventricular remodeling and heart failure following STEMI (57, 58) Reduction in hsCRP and IL-6 following NSTEMI
Canakinumab	Fully human monoclonal antibody against IL-1β	Neutralizing IL-1β antibody reduces coronary thickening and vasospasm (34)	CANTOS: reduction in MI, stroke, and cardiovascular-related death (59)
MLN1202	Neutralizing monoclonal antibody against CC-chemokine receptor 2 (CCR2)	Reduced atherosclerotic plaque in hypercholesterolemic CCR2 knockout mice (60)	Reduction in hsCRP (61)
Methotrexate	Folic acid antagonist Reduced T-cell proliferation Reduced cytokine release Reduced expression of cell-surface adhesion molecules	Reduced atherosclerotic plaque size and intimal migration of macrophages in hypercholesterolemic rabbits (62)	CIRT: no reduction in hsCRP, IL-1β, or IL-6; no reduction in cardiovascular events (63)

*IL, interleukin; CRP, C-reactive protein; hsCRP, high-sensitivity CRP; MI, myocardial infarction; STEMI, ST-segment elevation; MI; NF-κB, nuclear factor-kappa B; NSTEMI, non-ST-segment elevation MI; TNF-α, tumor necrosis factor-alpha*.

## Strategies to Target Residual Inflammatory Risk

### Lipid Lowering Therapy

**Figure 2:**
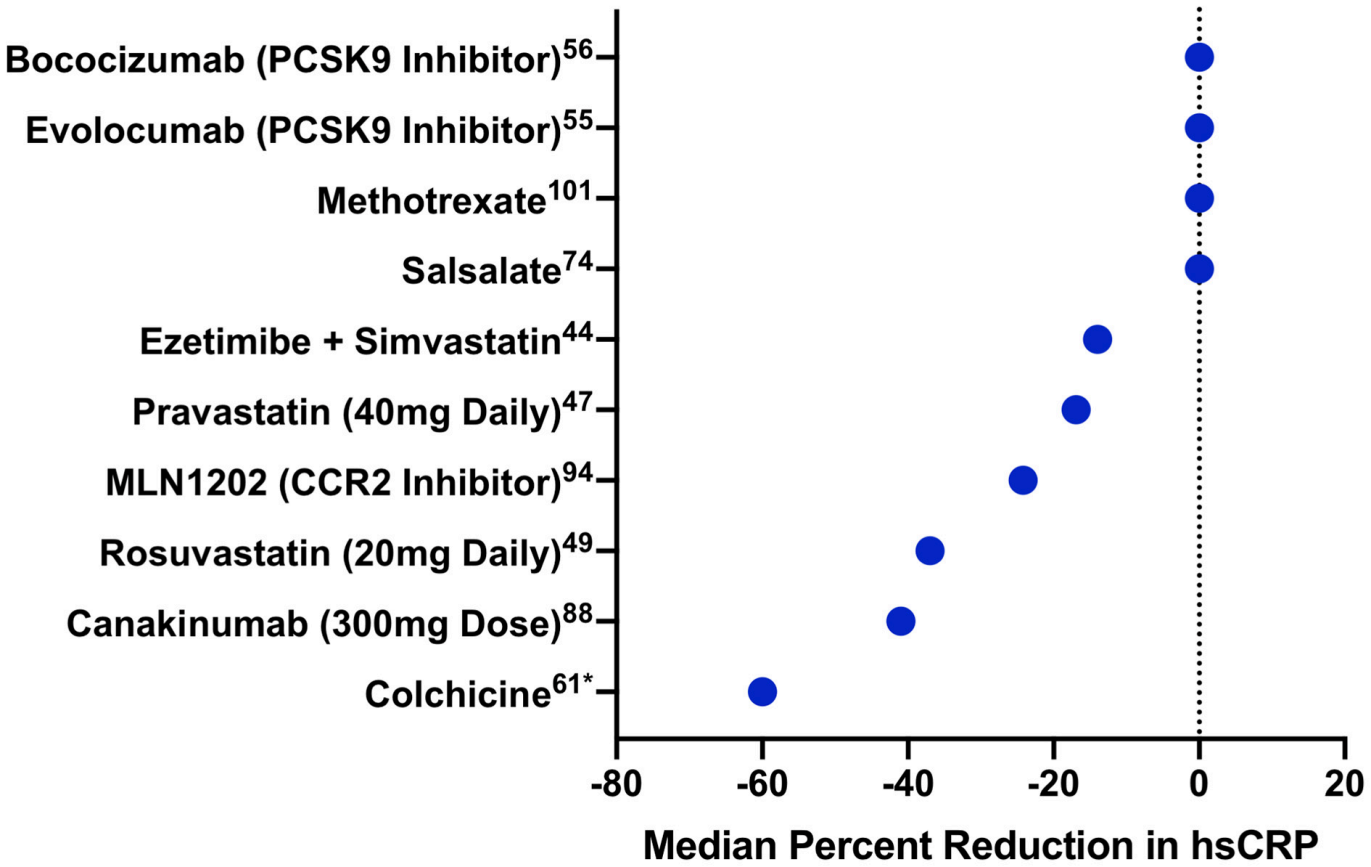
Median percentage change in hsCRP with different cardiovascular drugs. Median percentage change in hsCRP is displayed for several drugs from trials of patients either with established cardiovascular disease or at high risk for cardiovascular events. Drugs are ordered by their impact on hsCRP. PCSK9 indicates proprotein convertase subtilisin-kexin type 9; CCR2, CC-chemokine ligand 2 receptor. *Mean, rather than median, percentage change.

Several clinical trials have shown that statins reduce circulating markers of inflammation (Figure 2). The CARE study randomized 4,159 individuals with a history of MI and LDL-C levels 115–175 mg/dL to either pravastatin 40 mg daily or placebo (64). Among 472 randomly selected trial participants, pravastatin led to a 21.6% reduction in hsCRP over 5 years compared to treatment with placebo (65). In the PRINCE study, 1,702 patients free of cardiovascular disease were randomized to either pravastatin 40 mg daily or placebo. Again, statin therapy resulted in a 16.9% reduction in hsCRP at 24 weeks independent of LDL-C reduction (65). Lovastatin has a similar impact on hsCRP (66).

More intensive statin therapy leads to even further reduction of hsCRP. In JUPITER, rosuvastatin 20 mg daily reduced median hsCRP by 37% (*p* < 0.0001) compared to placebo (67). Similarly, in PROVE-IT TIMI 22, 57.5% of individuals treated with atorvastatin 80 mg daily achieved an hsCRP <2 mg/L compared to 44.9% of those treated with pravastatin 40 mg daily (68). Additional studies have found shown similar effects with intensive statin therapy (69, 70).

Other classes of lipid-lowering drugs have also been studied in terms of their impact on hsCRP reduction. In IMPROVE-IT, the combination of simvastatin and ezetimibe led to a 14% reduction in hsCRP (*p* < 0.001) compared to simvastatin alone over the duration of the trial (42). Although data on fibrates are limited, a meta-analysis of 1,635 patients treated with fibrates showed a 0.47 mg/L mean reduction in hsCRP compared to placebo (*p* = 0.046) (71). Notably, trials of PCSK9 inhibitors have not shown an impact on hsCRP despite pronounced reductions in LDL-C. FOURIER randomized 27,564 individuals with stable atherosclerotic disease and an LDL-C ≥70 mg/dL despite statin therapy to either evolocumab, a PCSK9 monoclonal antibody, or placebo (72). Following a median of 2.2 years, evolocumab had no significant impact on hsCRP (median reduction 0.2 mg/L) (73). Bococizumab, another monoclonal antibody against PCSK9, similarly had no impact on hsCRP reduction (74).

Statin therapy is not only beneficial in terms of hsCRP reduction but also reduction in cardiovascular event rates among those at high inflammatory risk. In PROVE-IT TIMI 22, those who achieved hsCRP levels <2 mg/L had fewer cardiovascular events independent of LDL-C lowering (39). The combination of simvastatin and ezetimibe yielded similar results in IMPROVE-IT (42). In JUPITER, the magnitude of hsCRP reduction achieved with rosuvastatin was proportional to the reduction in cardiovascular risk; those who achieved hsCRP levels <2 mg/L had a 55% reduction in the primary endpoint compared to those who achieved hsCRP levels ≥2 mg/L (*p* = 0.007) (67). Of note, the correlation between achieved LDL-C and hsCRP measures in JUPITER was minimal (*r* = 0.10), suggesting independence of these pathways (67). Additionally, among 6,766 participants in JUPITER, the genetic variants that achieved genome-wide significance for LDL-C reduction with rosuvastatin therapy were not associated with hsCRP reduction (75). These lines of evidence further support the notion of inflammatory risk independent of LDL-related risk in these patients.

### Colchicine

Although statins and ezetimibe both reduce hsCRP, researchers have long searched for a drug that could target inflammation independent of an impact on LDL-C. Based on both preclinical and clinical data, colchicine emerged as a potential candidate. The plant source of colchicine, the autumn crocus, has been recognized for centuries for its medicinal properties, and colchicine is commonly used to treat gout as well as acute pericarditis. Colchicine has several possible anti-inflammatory mechanisms of action. It inhibits cytoskeletal microtubule polymerization, which in turn inhibits vesicular secretion of inflammatory signals (76). In addition, colchicine may prevent activation of the NLRP3 inflammasome by cholesterol crystals, which in turn reduces release of IL-1β (77).

In a hyperlipidemia rat model, 2 weeks of therapy with colchicine led to lower plasma CRP levels than therapy with atorvastatin (4.03 mg/L vs. 5.35 mg/L, *p* < 0.05) (44). A pilot study of 200 patients with clinically stable coronary artery disease already receiving atorvastatin showed that 4 weeks of colchicine therapy led to a 60% decrease in mean hsCRP (45). Among 40 patients with ACS undergoing coronary angiography, circulating levels of the inflammatory markers IL-1β, IL-18, and IL-6 sampled at the coronary sinus were significantly elevated compared to peripheral levels, suggesting local release from the coronary arterial bed (46). Two doses of colchicine (1 mg initially followed by 0.5 mg 1 h later) led to statistically significant local reductions in these biomarkers relative to peripheral levels (*p* < 0.05 for all biomarkers).

Ultimately, the LoDoCo study tested colchicine therapy in a randomized trial setting. In this observer-blinded trial of 532 patients with stable coronary disease on optimal medical therapy, patients were randomized to colchicine 0.5 mg daily vs. placebo with a planned median follow-up of 3 years (78). Overall, colchicine therapy led to a 67% reduction in the composite endpoint of ACS, out-of-hospital cardiac arrest, and non-cardioembolic ischemic stroke. Of note, 11% of patients in the colchicine arm withdrew in the first month of treatment due to gastrointestinal side effects of the drug. Two large, phase 3 fully blinded placebo trials of colchicine in secondary prevention of cardiovascular disease, LoDoCo2 and COLCOT, are currently recruiting participants.

### Allopurinol

The link between plasma uric acid levels and cardiovascular disease remains controversial. In the Framingham Heart Study, investigators found no association between blood uric acid levels and incident cardiovascular disease after adjusting for traditional cardiovascular risk factors (79). However, additional prospective epidemiologic cohorts have shown an association between elevated uric acid levels and cardiovascular mortality after adjusting for traditional risk factors (80, 81). Data also suggest that uric acid crystals characteristically seen in gout can also activate the NLRP3 inflammasome (77). Allopurinol is a purine analog that inhibits xanthine oxidase, leading to urinary excretion of uric acid. It is commonly used to treat hyperuricemia in gout and other conditions. In a hypercholesterolemic ApoE knockout mouse model, treatment with allopurinol reduced aortic atheroma formation, foam cell development, and release of inflammatory cytokines (47). In a case-control study of 2,277 patients who suffered a first MI, allopurinol did not lead to a reduction in recurrent MI (48). In a study of 40 patients with acute ST elevation MI who were undergoing primary percutaneous coronary revascularization, 1 month of allopurinol therapy led to a 13% reduction in cardiovascular events (*p* = 0.002) compared to placebo (49). More recently, in a double-blind non-inferiority trial, investigators randomized 6,190 patients with gout and cardiovascular disease to either allopurinol or febuxostat, a non-purine xanthine oxidase inhibitor (82). Febuxostat was non-inferior in terms of reducing cardiovascular events, although data on changes in hsCRP or other inflammatory markers are unavailable. It remains unclear whether allopurinol modulates cardiovascular inflammatory risk independent of hyperuricemia, and to our knowledge, there are no ongoing clinical trials addressing this issue.

### Salicylates

Signaling through the nuclear factor-kappa B (NF-κB) pathway upregulates several inflammatory molecules, including IL-1 and IL-6 (83). Sodium salicylate and its prodrug, salsalate, are known to inhibit NF-κB activity (50, 83). In preliminary studies of both healthy individuals and young adults with obesity, salsalate therapy led to significant reductions in hsCRP (84, 85). However, in the follow up TINSAL-T2D study of 108 patients with type 2 diabetes mellitus, 48 weeks of salsalate had no impact on hsCRP levels compared to placebo (50). In an ancillary study of 75 patients from the overall trial (TINSAL-FMD), there was also no difference in flow-mediated dilation, a marker of endothelial dysfunction (51). More recently, in TINSAL-CVD, 257 patients with stable coronary artery disease were randomized to 30 months of salsalate or placebo (52). At 30 months, there was no difference in hsCRP or coronary plaque volume measured by computed tomography angiography. It remains unclear whether sodium salicylate or salsalate are useful in preventing clinical cardiovascular events.

### IL-6 Modulation

IL-6 inhibitors, such as tocilizumab, are important therapeutic options in autoimmune diseases such as rheumatoid arthritis and giant cell arteritis. Given the role of IL-6 in atherosclerosis, investigators have begun to examine the impact of IL-6 blockade in cardiovascular disease. Several studies have estimated the cardiovascular impact of IL-6 blockade with tocilizumab by using large-scale studies of genetic variants in the IL-6 receptor gene (*IL6R)* similarly associated with reduced IL-6 signaling. In one such study of 126,198 cases and controls of European ancestry, a single nucleotide polymorphism in *IL6R* associated with reduced receptor function also led to a decreased risk of coronary events (per allele odds ratio 0.95; 95% CI, 0.93–0.97) (24). Similarly, in a phenome-wide association study among 332,799 US veterans, this same variant was also associated with a reduction in both atherosclerotic disease and ischemic heart disease (odds ratio 0.95; 95% CI, 0.94–0.97) (86).

In a randomized, double-blind, placebo-controlled trial, 117 individuals with an acute non-ST elevation MI received either a single dose of tocilizumab or placebo (53). Tocilizumab led to more rapid plasma clearance of hsCRP measured by area under the curve (*p* < 0.001). Within this same trial, however, tocilizumab had no impact on coronary flow reserve at 6 months (54). The recently completed ENTRACTE trial (ClinicalTrials.gov NCT01331837) randomized 3,080 individuals with rheumatoid arthritis to either tocilizumab or etanercept, a TNF-α inhibitor, in a non-inferiority study design with a primary outcome of non-fatal MI, non-fatal stroke, or cardiovascular-related death; the publicly available results show tocilizumab was non-inferior to etanercept in terms of the primary outcome (55). Of note, cardiovascular events were rare with 2 ACS events and 16 acute MIs in the trial. More recently, sarilumab, another monocloncal antibody against the IL-6 receptor, has been shown to effectively reduce levels of hsCRP, but its impact on reducing cardiovascular events is unclear (87). The potential role of IL-6 inhibitors in preventing cardiovascular events may be limited by opportunistic infections as well as their known side effect of raising LDL-C (56). Nonetheless, there are ongoing discussions regarding future trials of IL-6 inhibitors in cardiovascular disease.

### IL-1 Modulation

As previously discussed, IL-1 is an attractive drug target for inflammation in cardiovascular disease due to its direct role in transducing inflammasome activation as well as its position upstream of not only IL-6 but multiple other inflammatory mediators. Several drugs that modulate IL-1 signaling are currently available. Anakinra is a humanized monoclonal antibody that decreases signaling via both IL-1α and IL-1β and is commonly used to treat rheumatoid arthritis (88). In small pilot studies of patients with acute ST segment elevations MIs, treatment with anakinra was associated with improved imaging measures of left ventricular remodeling (57) as well as a lower incidence of heart failure at 14 weeks (58). A randomized, placebo-controlled phase II study of 182 patients following non-ST elevation MI found that 14 days of anakinra therapy led to statistically significant reductions in both hsCRP and IL-6 (89). An ongoing trial is also assessing the impact of anakinra on hsCRP levels following ST elevation MI (90). However, in a Mendelian randomization study of 746,171 individuals, genetic variants upstream of the IL-1 receptor antagonist gene, which are associated with reduced IL-1 signaling, were also associated with increased levels of LDL-C and increased risk of both coronary heart disease and abdominal aortic aneurysm (29).

Canakinumab, a fully human IL-1β neutralizing monoclonal antibody, emerged as a potentially more viable candidate both because of the specific role IL-1β signaling plays in potentiating atherosclerosis and because it would leave host immune function via IL-1α intact. It had previously been used to treat Cryopyrin-Associated Periodic Syndromes, such as Muckle-Wells Syndrome, and there were limited data on its utility in cardiovascular disease. However, pilot data in 556 high risk patients with diabetes showed that canakinumab reduced hsCRP, fibrinogen, and IL-6 with no impact on atherogenic lipids (91).

Ultimately, investigators launched the Canakinumab Anti-inflammatory Thrombosis Outcome Study (CANTOS) to further test this drug as a secondary prevention tool (92). CANTOS was a randomized, double-blind, placebo-controlled study of 10,061 patients with a history of MI and residual inflammatory risk defined as hsCRP levels ≥2 mg/L. Investigators used three different subcutaneous doses of canakinumab: 50, 150, and 300 mg administered every 3 months. The primary endpoint was a composite of non-fatal MI, non-fatal stroke, or cardiovascular death (MACE), and the pre-specified secondary endpoint was MACE plus hospitalization for unstable angina requiring urgent revascularization (MACE+).

Within the trial, median follow-up was 3.7 years. Approximately 93% of participants were on lipid lowering therapy at baseline (59). Mean baseline LDL-C was 82 mg/dL, and median baseline hsCRP was 4.2 mg/L. Canakinumab was effective at reducing both hsCRP and IL-6 in a dose-dependent manner. Following 48 weeks of treatment, median hsCRP decreased 26–41%, and median IL-6 decreased 19–38% at 12 months. There was no change in LDL-C with canakinumab.

Treatment with the higher 150 or 300 mg doses of canakinumab led to a 15% reduction in the primary endpoint (*p* = 0.007), although there was no significant change in MACE with the 50 mg dose (59). Higher doses of canakinumab also led to a 17% reduction in MACE+ (*p* = 0.0006). There was no evidence of overall differences in efficacy of canakinumab based on sex, age, diabetes, smoking, body-mass index, or baseline levels of cholesterol or hsCRP (93). However, in a pre-specified analysis, more pronounced reductions in hsCRP with canakinumab led to more significant reductions in cardiovascular events (93). Study participants with on-treatment hsCRP levels <2 mg/L had a 25% reduction in MACE (*p* < 0.0001) compared to only 5% among those with on-treatment hsCRP levels ≥2 mg/L. Similarly, reduction in hsCRP <2 mg/L also led to a 31% reduction in cardiovascular death (*p* = 0.0004) and all-cause mortality (*p* < 0.0001) compared to non-significant reductions with hsCRP levels ≥2 mg/L. Similar on-treatment data have been presented from CANTOS for IL-6 (94).

Overall, canakinumab was safe and well-tolerated. Although rates of death due to infection or sepsis were low, there was a slightly higher incidence of approximately one per thousand among those treated with canakinumab vs. placebo (incidence rate 0.31 vs. 0.18 per 100 person-years, *p* = 0.02). Of the different types of infections observed, only pseudomembranous colitis occurred more frequently in the canakinumab group. There were no instances of opportunistic infection, again suggesting that the innate immune system remained intact while receiving canakinumab. Additional trials of other IL-1β inhibitors as well as drugs that target the NLRP3 inflammasome are currently under consideration.

### Chemokine Modulation

Beyond IL-1 and IL-6, several other chemokines are involved in atherosclerosis. One such molecule is monocyte chemoattractant protein-1 (MCP-1), or CC-chemokine ligand 2 (CCL2). MCP-1 signaling via its receptor, CCR2, is responsible for monocyte activation and infiltration as part of the inflammatory response (95). CCL2 is also present in atherosclerotic plaques (95). In hyperlipidemia ApoE and CCR2 double knockout mice, there is a marked reduction in atherosclerotic plaque with no change in lipid measures compared to ApoE knockout mice (60). Among 2,270 patients with ACS in the OPUS-TIMI 16 trial, plasma MCP-1 levels above the 75th percentile were associated with a 53% increased risk of death or MI after 10 months of follow-up even after adjusting for traditional cardiovascular risk factors and CRP (96). In a double-blind, randomized controlled trial of 106 adults with at least 2 cardiovascular risk factors and hsCRP levels >3.0 mg/L, participants were treated with either MLN1202, a neutralizing monoclonal antibody against CCR2, or placebo (61). Following 12 weeks of treatment, MLN1202 led to a significant median percent change in hsCRP compared to placebo (−24.2% vs. 2.5%, *p* = 0.009).

### Methotrexate

Methotrexate is a folic acid antagonist originally developed as a chemotherapeutic drug. However, in low doses, it is also an effective, safe, and well-tolerated anti-inflammatory agent commonly used to treat rheumatoid arthritis and psoriasis. Although the mechanisms of its anti-inflammatory effects are unclear, data suggest it reduces T-cell proliferation, secretion of cytokines, and matrix metalloproteinases, and expression of adhesion molecules by altering adenosine metabolism, all of which make it an attractive candidate in atherosclerotic disease (76, 97). Preclinical models further support this notion; in a hypercholesterolemia rabbit model, intravenous methotrexate reduced both atherosclerotic plaque size and subintimal migration of macrophages compared to placebo (62).

Observational data also support the role of methotrexate in preventing cardiovascular disease. In a cross-sectional study of 4,363 patients with rheumatoid arthritis, prolonged exposure to methotrexate was associated with a 15% reduction in cardiovascular events (98). Within a prospective Danish cohort of 2,400 patients with severe psoriasis, methotrexate was associated with a 50% reduction in a composite endpoint of cardiovascular-related death, MI, and stroke (99). Subsequently, in a systematic review of 10 observational studies of patients receiving methotrexate for rheumatologic diseases, treatment was associated with a 21% reduced risk of overall cardiovascular disease and an 18% reduced risk of MI (100).

On the strength of these data, investigators launched the National Heart, Lung, and Blood Institute-sponsored Cardiovascular Inflammation Reduction Trial (CIRT) in 2012 (101). CIRT was a randomized, double-blind, placebo-controlled trial to determine whether low-dose methotrexate would lead to a reduction cardiovascular events. Eligible individuals included those ≥18 years of age with a history of MI or multi-vessel coronary artery disease as well as type 2 diabetes mellitus or metabolic syndrome. Baseline measurement of hsCRP or other inflammatory markers was not required to determine eligibility.

Following a 4-week active run-in period, those randomized to methotrexate therapy received 15 mg orally per week, and this increased to 20 mg orally per week after a 4 month period if tolerated. All patients also received a folic acid supplement. Individuals with a history of malignancy, liver disease, kidney disease, chronic infection, or pre-existing rheumatologic disease were excluded from the study, and a centralized team of rheumatologists monitored all participants to ensure safety and coordinate temporary interruptions or adjustments in the study drug according to guidelines set forth by the American College of Rheumatology. The primary endpoint was a composite of non-fatal MI, non-fatal stroke, hospitalization for unstable angina requiring urgent revascularization, and cardiovascular death.

The trial was terminated early upon the recommendation of the data and safety monitoring board after the study met a prespecified threshold for futility of low-dose methotrexate therapy on the primary outcome (63). At the time of study termination, 66.1% of the target study population had begun open-label run-in, and 51.3% of the target population had undergone study drug randomization. Vital status was available on nearly all participants with only 10 patients ultimately lost to follow-up.

Within the trial, median age was ~66 years, and 19% of participants were female (63). 61% of study participants qualified for enrollment due to a prior MI, and 39% qualified due to a history of multivessel coronary disease. Eighty-six percentage were taking a statin at baseline, and 86% were receiving antiplatelet or antithrombotic therapy. Median baseline LDL-C was 68.0 mg/dL, and median hsCRP was normal at 1.5 mg/L.

Laboratory analyses at 8 months following randomization showed that low-dose methotrexate had no significant impact on blood levels of hsCRP, IL-6, or IL-1β (63). The lack of impact on hsCRP extended to 2 years following randomization. After a median follow-up of 2.3 years, with a maximum follow-up of 5 years, methotrexate therapy had no impact on the primary endpoint (hazard ratio 0.96; 95% CI, 0.79–1.16). Similarly, no differences were seen in terms of cardiovascular death (hazard ratio 1.14; 95% CI, 0.76–1.72) or all-cause mortality (hazard ratio 1.16; 95% CI, 0.87–1.56). There was also no difference in the effect of methotrexate based on baseline hsCRP level. As such, taken together, the positive CANTOS trial and the informative but neutral CIRT trial indicate that inhibition of the central IL-1β to IL-6 to CRP pathway is likely a requirement for cytokine-mediated atheroprotection.

In terms of safety, methotrexate was well-tolerated with no differences in serious adverse events or serious infections (63). As shown in other studies, methotrexate was associated with modest leukopenia as well as mild increases in alanine and aspartate aminotransferase concentrations. There was also an increased risk of cancer with methotrexate therapy, although this was driven primarily by rates of non-basal-cell skin cancers.

Given the results of CIRT, low-dose methotrexate may not have a role in the reduction of cardiovascular events outside of a patient population with rheumatologic disease. Of note, patients enrolled in CIRT did not have residual inflammatory risk at baseline, as evidenced by normal baseline hsCRP levels. However, this may not fully account for the trial outcome since methotrexate therapy did not further reduce inflammatory biomarkers regardless of the baseline hsCRP. This lack of impact on inflammatory biomarkers, in contrast to the results from CANTOS, suggests that broadly targeting inflammation in cardiovascular disease may be insufficient. Rather, it may be necessary to target specific pathways within the broader inflammatory response. Ancillary studies currently underway are also examining the impact of methotrexate on coronary flow reserve as well as peripheral artery disease, and these may provide additional insight into the role of inflammation in cardiovascular disease.

## Future Directions

With the results of CANTOS, we now have evidence from bench to bedside of inflammation's role in atherosclerotic disease, and proof-of-principle for the inflammatory hypothesis of atherothrombosis. As CIRT highlighted, however, targeting all inflammatory pathways may not be successful in reducing cardiovascular events, and it appears signaling in the NLRP3 inflammasome➔IL-1➔IL-6 pathway is the most promising for future drug development. Although there are important discussions surrounding cost, safety, and patient selection for these emerging therapies, many of these concerns will be tempered with time as additional therapeutic options are developed. Ultimately, clinical trials like those discussed in this review and in other spheres of cardiovascular drug development will hopefully lead to an individualized, tailored approach to address all forms of residual cardiovascular risk.

## Author Contributions

AA was responsible for conceptualization and drafting of the manuscript. PR was responsible for conceptualization and editing of the manuscript.

### Conflict of Interest Statement

PR served as the Principal Investigator of CANTOS and received research grant support to conduct CANTOS from its sponsor, Novartis. PR also served as the Trial Co-Chair for the SPIRE studies of bococizumab and received research grant support to conduct these trials. PR also served as the Principal Investigator of CIRT. PR has also served as a consultant to Novartis, Pfizer, Merck, Amgen, and Sanofi, and is listed as a co-inventor on patents held by the Brigham and Women's Hospital that relate to the use of inflammatory biomarkers in cardiovascular disease and diabetes that have been licensed to AstraZeneca and Siemens. The remaining author declares that the research was conducted in the absence of any commercial or financial relationships that could be construed as a potential conflict of interest.
